# Literary Thefts

**Published:** 1887-10

**Authors:** 


### LITERARY THEFTS.

We are continually annoyed by the appropriation, by some of our exchanges, of entire articles original in this journal, without a word or sign of credit. This is not only unfair to us, but also to the author, whose name should accompany its transference into other channels.

We do not copyright the JOURNAL, because we desire that its contents may be made to serve the public in many ways, but we certainly do expect that other publications, which make use of them, will do us the justice to mention the source from which they obtained them. This is all we ask, and none will dispute that it is little enough.

Not long ago we saw one of our small poems in a country sheet, under the head of "Floating." It is evident that this paper so found it in some other sheet. Again, only recently, a Southern medical journal published an entire article, from the pen of the writer, on its first page, as if it were original there, and now to-day, we find in a Delaware exchange a little translation from the German entitled "The Maid's Behest," published in our June number. But the paper in question changed the title to that of "The Moon" and gave no credit either to its German author or to HALL'S JOURNAL, wherein it is presumed it was found and appropriated, after being shorn of some of its appendages.

We wish to say to our exchanges, one and all, that they are welcome to republish any articles found in our columns, by giving the JOURNAL credit for them. It is our unvarying custom to give credit for all articles transferred from other publications to ours, and we ask that a like courtesy be extended to us by our contemporaries, wherever located

